# Trimetazidine Use in Parkinson’s Disease: Is It a Resolved Problem?

**DOI:** 10.1523/ENEURO.0452-20.2021

**Published:** 2021-05-14

**Authors:** Dávid Pintér, Dániel Bereczki, András Ajtay, Ferenc Oberfrank, József Janszky, Norbert Kovács

**Affiliations:** 1 Department of Neurology, Medical School, University of Pécs, Pécs, Hungary 7623; 2 Department of Neurology, Medical School, Semmelweis University, Budapest, Hungary 1085; 3 Hungarian Academy of Sciences - Semmelweis University, Neuroepidemiology Research Group, Budapest, Hungary; 4 Institute of Experimental Medicine, Budapest, Hungary 1083; 5 Hungarian Academy of Sciences - University of Pécs, Clinical Neuroscience Magnetic Resonance Research Group, Pécs, Hungary 7623

**Keywords:** trimetazidine, Parkinson’s disease, angina pectoris, European Medicines Agency, interrupted time series analysis

## Abstract

Trimetazidine (TMZ), an antianginal drug, can worsen the symptoms of movement disorders, therefore, the European Medicines Agency (EMA) recommended avoiding the use of this drug in Parkinson’s disease (PD). We investigated the impact of this recommendation on the observed trend of TMZ use in PD in Hungary from 2010 to 2016 by conducting a nationwide, retrospective study of health administrative data of human subjects. Interrupted time series analyses were performed to explore changes in user trends after the EMA recommendations. We found that TMZ use in PD decreased by 6.56% in each six-month interval after the EMA intervention [a change in trend of −530.22, 95% confidence interval (CI) = −645.00 to −415.44, *p* < 0.001 and a decrease in level of −567.26, 95% CI = −910.99 to −223.53, *p* = 0.005 12 months postintervention]. TMZ discontinuation was the highest immediately after the intervention, however, its rate slowed down subsequently (a change in trend of −49.69, 95% CI = −85.14 to −14.24, *p* = 0.11 without significant level effects). The rate of new TMZ prescriptions did not reduce significantly, therefore, the decreased overall use was mainly attributable to the increased rate of discontinuation only. The main indications for TMZ use were circulatory system disorders, especially angina pectoris, however, off-label utilization was also considerable (40%). The EMA recommendations on TMZ use seem to be only moderately effective in Hungary. Although the number of patients with PD on the drug modestly decreased after the EMA restrictions, TMZ is still widely used in PD for both on-label and off-label indications.

## Significance Statement

Trimetazidine (TMZ) can worsen the symptoms of movement disorders in a clinically relevant manner and its use is consequently not recommended in Parkinson’s disease (PD) by the European Medicines Agency (EMA). The impact of the EMA recommendations on TMZ use in PD has not yet been evaluated, therefore, we conducted a nationwide, retrospective study to address this question in Hungary. According to our results, the restrictions on TMZ use are only moderately effective. Although the number of patients with PD on the drug modestly decreased after the EMA recommendations, TMZ is still widely used in PD for both on-label and off-label indications. Our findings promote another safety communication to resolve a clinically important problem and to improve the management of patients with PD.

## Introduction

Trimetazidine (TMZ), a widely used antiischemic drug in Europe, is usually prescribed as a long-term treatment for angina pectoris (cardiological indication), and in some countries for tinnitus, vertigo/dizziness (otological indications), and visual disturbances (ophthalmological indications). Because medicines containing TMZ had been reported both causing reversible parkinsonism, tremor, and orofacial dyskinesia (Martí Massó, 2004; Martí Massó et al., 2005; Masmoudi et al., 2005; Sommet et al., 2005; Sivet et al., 2008), and worsening the symptoms of existing movement disorders such as Parkinson’s disease (PD; Martí Massó et al., 2005), the French National Pharmacovigilance Commission recommended the reevaluation of the role of TMZ in antianginal treatment on May 19, 2009 (Commission nationale de pharmacovigilance, 2009). The results of this safety analysis led to the suspension of the French authorization of TMZ on April 7, 2011 (Réunion de la Commission d’AMM du 7 avril 2011, 2011). Because of the concerns of the French medicines regulatory agency over the safety and efficacy of TMZ, the European Medicines Agency (EMA) also reviewed the benefits and risks of the drug between April 22, 2011 and June 22, 2012 (European Medicines Agency, 2012a). After the review, the drug was delicensed as a treatment option for tinnitus, vertigo, and vision disturbances, and prescription of TMZ became contraindicated in patients having PD or severely reduced kidney function (European Medicines Agency, 2012a). Furthermore, TMZ has only been recommended as a second-line treatment for angina pectoris in accordance with the EMA restrictions and recent guidelines for the management of chronic coronary syndromes (European Medicines Agency, 2012a; Knuuti et al., 2020).

Although other antianginal medications with similar level of evidence are also available as second-line treatments (Danchin et al., 2011; Knuuti et al., 2020), TMZ has remained to be one of the most frequently used agents in the symptomatic treatment for angina pectoris (Ponikowski et al., 2016; Pintér et al., 2019). Furthermore, its use in patients with PD also seems to remain extensive (Kwon et al., 2019; Pintér et al., 2019) despite published warnings concerning TMZ treatment and the clear recommendation against the prescription of TMZ in movement disorders.

Postmarketing safety analyses of available drugs have an essential role in reaching and maintaining high-quality patient care. In the European Union, both national pharmacological agencies and the EMA have their pharmacovigilance services to monitor drug safety. Recently, numerous safety warnings have been made by international regulatory agencies for various neurologic agents. Similarly to TMZ in PD, the EMA took regulatory actions for valproic acid (VPA) use in girls, women of childbearing age, and pregnant females based on postmarketing data. Although the efficacy of the restrictions on VPA use by the EMA has been thoroughly evaluated (European Medicines Agency, 2014; Vajda et al., 2014; Wen et al., 2015; Liu et al., 2017; Karlsson Lind et al., 2018; Kinney et al., 2018; Virta et al., 2018; Jacob et al., 2019; Puteikis et al., 2019), only little efforts have been made to generate such information concerning the impact of the EMA warning on the clinical practice with TMZ thus far (von Bredow et al., 2018). Therefore, we conducted a study in Hungary, a country in the European Union with a population of ∼10 million of which ∼400,000 inhabitants suffered from stable coronary heart disease in 2018 (Pintér et al., 2019). Our aims were as follows: (1) to determine, whether there is any change in the trend of TMZ use among patients with PD afterthe EMA recommendations; (2) to compare trends of TMZ discontinuation in PD before and after the EMA restrictions; (3) to compare trends of new TMZ prescriptions among PD patients before and after the EMA regulatory intervention; and (4) to explore the indications for ongoing TMZ treatment and new prescriptions in the PD population.

## Materials and Methods

### Study design

A nationwide, retrospective study of anonymized health care administrative data of both male and female human subjects was conducted to assess the effectiveness of the EMA regulatory event on TMZ use. The data evaluated in this study was obtained from the database of the National Health Insurance Fund of Hungary, a country with a single-payer health insurance system. In this database, data on drug utilization regardless of being prescribed by state-funded or private services has been recorded since 2000 (Gresz, 2012). In respect of drug prescription refills, not only the social security numbers of patients but also data on the type and the dose of medications and the indications for prescriptions, that are indicated by the International Classification of WHO Diseases, 10th Revision, Clinical Modification (ICD-10-CM) codes, are strictly recorded on an individual level. In addition, the database includes relevant data on both outpatient and inpatient care, therefore, it is suitable for detecting chronic medication use. Because both the reimbursement for medications and the funding of hospital care are performed based on these reports, this database is a reliable representation of data of patients in the Hungarian health care. Original patient identifiers were anonymized, and the encrypted patient identifier was used for linking medical information to prescription refills.

The study design was similar to that used by Puteikis et al. (2019), to assess the impact of the EMA regulation on VPA use. To evaluate changes in the numbers of PD patients treated with TMZ, new prescriptions on TMZ and withdrawal of the drug in PD over time, the analysis of an interrupted time series model was applied. This method has previously been described in more detail elsewhere (Ramsay et al., 2003; Bernal et al., 2017).

### Study data

In the first analysis aiming to evaluate the change in overall TMZ use in PD, patient reimbursement information for TMZ [Anatomical Therapeutic Chemical (ATC) code C01EB15] was used from 2010 to 2016. A total of 464,116 subjects treated with TMZ in this period were identified. Only patients aged older than 18 years at the initiation of TMZ, having the diagnosis of PD (ICD-10-CM code G20), treated with antiparkinsonian medications (ATC code N04), as a confirmation of the diagnosis of PD, and with concomitant TMZ use were finally included in this analysis. We analyzed data for every half-year because according to the EMA recommendation, there had been no need for urgent intervention, changes in treatment introduced at the “next routine appointment” had been acceptable (European Medicines Agency, 2012a). To eliminate the effect of death on our results, data of patients who had died in the half-year examined was excluded. The outcome was the number of patients in the different half-years, and the date of the end of the EMA assessment procedure (June 22, 2012) was the selected intervention point.

In another analysis, we examined the frequency of new TMZ prescriptions and TMZ discontinuation in PD between 2010 and 2016. To include data of a patient, the following criteria must have been met: (1) age older than 18 years at the initiation of TMZ; (2) the diagnosis of PD (ICD-10-CM code G20); and (3) treatment with antiparkinsonian medications (ATC code N04). During the extraction of data of newly initiated patients, the diagnosis of PD must have been established before the first prescription of TMZ. With respect to TMZ discontinuation, efforts were made to eliminate the effects of death and intolerance to or ineffectiveness of TMZ on the results. Because patients are generally supplied with TMZ for 30 d with a prescription in Hungary, subjects with at least two consecutive prescriptions and consequently at least 60 d of treatment were considered as chronic TMZ users. Data for every half-year was evaluated in this subanalysis, and the date of the appearance of the EMA recommendations (22 June 2012) was used as an intervention point.

Finally, we attempted to identify the main indications for TMZ initiation and ongoing treatment in PD. Based on certain preselected ICD-10-CM codes, that were collected for each included subject, we made the following categorizations: (1) antianginal indication (ICD-10-CM code I20, on-label prescriptions); (2) all other cardiological indications (ICD-10-CM codes I00-I99 with the exemption of I20, possibly off-label indications); (3) ophthalmological indications (ICD-10-CM H30-H36, definitely off-label indications after the EMA warning); and (4) otological indications (ICD-10-CM codes H80-H83, definitely off-label indications after the EMA warning).

This study protocol was approved by the 7603-PTE.2018 Institutional and Regional Ethical Board. All study-related procedures were performed in accordance with the Helsinki Declaration of 1975.

### Statistical analysis

A non-seasonal autoregressive integrated moving average (ARIMA) model was used. All analyses were performed following the guidance provided by the Cochrane Effective Practice and Organization of Care Group (Cochrane Effective Practice and Organisation of Care, 2017).

The IBM SPSS software package (version 24.0.2, IBM Inc.) was used for all statistical analyses. The level of statistical significance was set at 0.05.

### Data availability

Because the Ethical Approval of the present study does not authorize the authors to publish the data, data are not made available.

## Results

**Figure 1.**
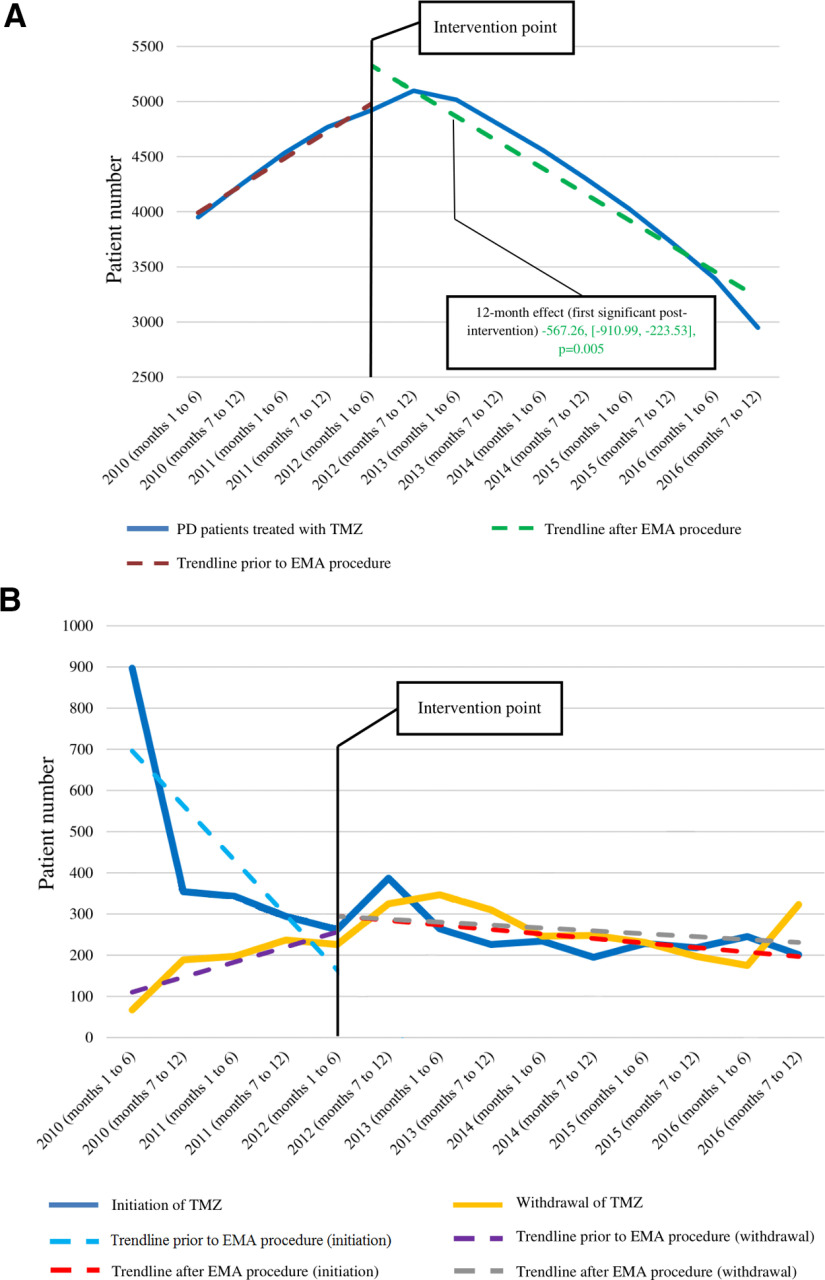
Patients having Parkinson’s disease with ongoing trimetazidine treatment (***A***), new initiations or withdrawal (***B***) from 2010 to 2016 and interrupted time series models. EMA, European Medicines Agency; PD, Parkinson’s disease; TMZ, trimetazidine.

The absolute number of PD patients treated with TMZ showed a gradual increase of an average of 260 in each six-month interval [95% confidence interval (CI) = 172.38–346.80, *p* < 0.001] before the EMA assessment procedure which means an average increase of 5.64% in each half-year. The overall TMZ use in PD reached its maximum (5098 patients) immediately after the intervention (the second half-year of 2012). Subsequently, the number of PD patients treated with the drug showed an average decrease of 6.56% (269 patients) in each six-month interval. According to the ARIMA model, there was a significant change in the preintervention trend of overall TMZ use in PD (−530.22, 95% CI = −645.00 to −415.44, *p* < 0.001). Additionally, we found a significant decrease in level delayed by 12 months (−567.26, 95% CI = −910.99 to −223.53, *p* = 0.005) and this effect remained significant during all subsequent postintervention six-month periods examined by this study. The relative 54-month effect was −57.93% (Fig. 1*A*; Table 1).

**Table 1:** ARIMA model parameters for Figure 1*A*

					Estimate	Standard error	*t* value	*p* value
ARIMA model parameters 1	Outcomes	No transformation	Constant		3726.399	145.993	25.525	<0.001
			AR	Lag 1	0.609	0.446	1.364	0.206
	Time period	No transformation	Numerator	Lag 0	259.585	38.550	6.734	<0.001
	Phase	No transformation	Numerator	Lag 0	3144.925	305.505	10.294	<0.001
	Interact	No transformation	Numerator	Lag 0	−530.221	50.743	−10.449	<0.001
ARIMA model parameters 2				
	Outcomes	No transformation	Constant		3725.494	145.369	25.628	<0.001
			AR	Lag 1	0.605	0.447	1.352	0.209
	Phase	No transformation	Numerator	Lag 0	−37.185	133.227	−0.279	0.786
	6 months preintervention	No transformation	Numerator	Lag 0	259.901	38.473	6.755	<0.001
	6 months postintervention	No transformation	Numerator	Lag 0	−270.593	29.031	−9.321	<0.001
ARIMA model parameters 3				
	Outcomes	No transformation	Constant		3725.852	145.951	25.528	<0.001
			AR	Lag 1	0.609	0.446	1.364	0.206
	Phase	No transformation	Numerator	Lag 0	−567.264	151.953	−3.733	0.005
	12 months preintervention	No transformation	Numerator	Lag 0	259.802	38.542	6.741	<0.001
	12 months postintervention	No transformation	Numerator	Lag 0	−270.729	29.187	−9.276	<0.001
ARIMA model parameters 4				
	Outcomes	No transformation	Constant		3725.668	145.560	25.595	<0.001
			AR	Lag 1	0.606	0.447	1.356	0.208
	Phase	No transformation	Numerator	Lag 0	−1097.990	183.443	−5.985	<0.001
	18 months preintervention	No transformation	Numerator	Lag 0	259.844	38.496	6.750	<0.001
	18 months postintervention	No transformation	Numerator	Lag 0	−270.607	29.076	−9.307	<0.001
ARIMA model parameters 5				
	Outcomes	No transformation	Constant		3724.868	145.799	25.548	<0.001
			AR	Lag 1	0.608	0.447	1.362	0.206
	Phase	No transformation	Numerator	Lag 0	−1629.819	222.141	−7.337	<0.001
	24 months preintervention	No transformation	Numerator	Lag 0	260.098	38.526	6.751	<0.001
	24 months postintervention	No transformation	Numerator	Lag 0	−270.753	29.171	−9.282	<0.001
ARIMA model parameters 6				
	Outcomes	No transformation	Constant		3724.800	145.744	25.557	<0.001
			AR	Lag 1	0.608	0.447	1.362	0.206
	Phase	No transformation	Numerator	Lag 0	−2160.452	264.907	−8.156	<0.001
	30 months preintervention	No transformation	Numerator	Lag 0	260.093	38.520	6.752	<0.001
	30 months postintervention	No transformation	Numerator	Lag 0	−270.776	29.142	−9.291	<0.001
ARIMA model parameters 7				
	Outcomes	No transformation	Constant		3725.820	145.699	25.572	<0.001
			AR	Lag 1	0.607	0.447	1.359	0.207
	Phase	No transformation	Numerator	Lag 0	−2688.893	309.975	−8.675	<0.001
	36 months preintervention	No transformation	Numerator	Lag 0	259.782	38.514	6.745	<0.001
	36 months postintervention	No transformation	Numerator	Lag 0	−270.635	29.101	−9.300	<0.001
ARIMA model parameters 8				
	Outcomes	No transformation	Constant		3725.288	145.465	25.609	<0.001
			AR	Lag 1	0.606	0.447	1.354	0.209
	Phase	No transformation	Numerator	Lag 0	−3220.464	356.342	−9.038	<0.001
	42 months preintervention	No transformation	Numerator	Lag 0	259.944	38.486	6.754	<0.001
	42 months postintervention	No transformation	Numerator	Lag 0	−270.626	29.045	−9.317	<0.001
ARIMA model parameters 9				
	Outcomes	No transformation	Constant		3725.283	145.604	25.585	<0.001
			AR	Lag 1	0.607	0.447	1.357	0.208
	Phase	No transformation	Numerator	Lag 0	−3751.680	404.167	−9.282	<0.001
	48 months preintervention	No transformation	Numerator	Lag 0	259.969	38.501	6.752	<0.001
	48 months postintervention	No transformation	Numerator	Lag 0	−270.673	29.106	−9.300	<0.001
ARIMA model parameters 10				
	Outcomes	No transformation	Constant		3725.515	146.026	25.513	<0.001
			AR	Lag 1	0.610	0.446	1.368	0.205
	Phase	No transformation	Numerator	Lag 0	−4282.119	453.283	−9.447	<0.001
	54 months preintervention	No transformation	Numerator	Lag 0	259.869	38.551	6.741	<0.001
	54 months postintervention	No transformation	Numerator	Lag 0	−270.905	29.197	−9.279	<0.001

TMZ discontinuation increased by 50.50% (40 patients) on average in each six-month preintervention period. Withdrawal of the drug was the highest (347 patients) in the second six-month period after posting the EMA recommendations (first half-year of 2013). In the postintervention period, the average increase in TMZ discontinuation was only 3.51% (11 patients) in each six-month interval. The ARIMA model globally detected a negative change in the preintervention trend of TMZ withdrawal (−49.69, 95% CI = −85.14 to −14.24, *p* = 0.11) without significant level effects. The relative 54-month effect was −62.69% (Fig. 1*B*; Table 2).

**Table 2:** ARIMA model parameters for TMZ withdrawal in Figure 1*B*

					Estimate	Standard error	*t* value	*p* value
ARIMA model parameters 1	Outcomes	No transformation	Constant		82.121	47.138	1.742	0.115
			AR	Lag 1	−0.343	0.567	−0.605	0.560
	Time period	No transformation	Numerator	Lag 0	34.983	14.450	2.421	0.039
	Phase	No transformation	Numerator	Lag 0	327.730	75.612	4.334	0.002
	Interact	No transformation	Numerator	Lag 0	−49.690	15.668	−3.171	0.011
ARIMA model parameters 2				
	Outcomes	No transformation	Constant		82.142	47.147	1.742	0.115
			AR	Lag 1	−0.343	0.567	−0.605	0.560
	Phase	No transformation	Numerator	Lag 0	29.606	55.124	0.537	0.604
	6 months preintervention	No transformation	Numerator	Lag 0	34.977	14.453	2.420	0.039
	6 months postintervention	No transformation	Numerator	Lag 0	−14.705	6.041	−2.434	0.038
ARIMA model parameters 3				
	Outcomes	No transformation	Constant		82.141	47.146	1.742	0.115
			AR	Lag 1	−0.343	0.567	−0.605	0.560
	Phase	No transformation	Numerator	Lag 0	−20.078	65.658	−0.306	0.767
	12 months preintervention	No transformation	Numerator	Lag 0	34.977	14.452	2.420	0.039
	12 months postintervention	No transformation	Numerator	Lag 0	−14.706	6.041	−2.434	0.038
ARIMA model parameters 4				
	Outcomes	No transformation	Constant		82.139	47.146	1.742	0.115
			AR	Lag 1	−0.343	0.567	−0.605	0.560
	Phase	No transformation	Numerator	Lag 0	−69.764	77.938	−0.895	0.394
	18 months preintervention	No transformation	Numerator	Lag 0	34.978	14.452	2.420	0.039
	18 months postintervention	No transformation	Numerator	Lag 0	−14.706	6.041	−2.434	0.038
ARIMA model parameters 5				
	Outcomes	No transformation	Constant		82.137	47.145	1.742	0.115
			AR	Lag 1	−0.343	0.567	−0.605	0.560
	Phase	No transformation	Numerator	Lag 0	−119.451	91.262	−1.309	0.223
	24 months preintervention	No transformation	Numerator	Lag 0	34.979	14.452	2.420	0.039
	24 months postintervention	No transformation	Numerator	Lag 0	−14.706	6.041	−2.434	0.038
ARIMA model parameters 6				
	Outcomes	No transformation	Constant		82.134	47.144	1.742	0.115
			AR	Lag 1	−0.343	0.567	−0.605	0.560
	Phase	No transformation	Numerator	Lag 0	−169.141	105.233	−1.607	0.142
	30 months preintervention	No transformation	Numerator	Lag 0	34.979	14.452	2.420	0.039
	30 months postintervention	No transformation	Numerator	Lag 0	−14.706	6.041	−2.434	0.038
ARIMA model parameters 7				
	Outcomes	No transformation	Constant		82.131	47.142	1.742	0.115
			AR	Lag 1	−0.343	0.567	−0.605	0.560
	Phase	No transformation	Numerator	Lag 0	−218.834	119.624	−1.829	0.101
	36 months preintervention	No transformation	Numerator	Lag 0	34.980	14.451	2.421	0.039
	36 months postintervention	No transformation	Numerator	Lag 0	−14.706	6.041	−2.435	0.038
ARIMA model parameters 8				
	Outcomes	No transformation	Constant		82.128	47.141	1.742	0.115
			AR	Lag 1	−0.343	0.567	−0.605	0.560
	Phase	No transformation	Numerator	Lag 0	−268.529	134.300	−1.999	0.077
	42 months preintervention	No transformation	Numerator	Lag 0	34.981	14.451	2.421	0.039
	42 months postintervention	No transformation	Numerator	Lag 0	−14.706	6.040	−2.435	0.038
ARIMA model parameters 9				
	Outcomes	No transformation	Constant		82.125	47.140	1.742	0.115
			AR	Lag 1	−0.343	0.567	−0.605	0.560
	Phase	No transformation	Numerator	Lag 0	−318.228	149.176	−2.133	0.062
	48 months preintervention	No transformation	Numerator	Lag 0	34.982	14.450	2.421	0.039
	48 months postintervention	No transformation	Numerator	Lag 0	−14.707	6.040	−2.435	0.038
ARIMA model parameters 10				
	Outcomes	No transformation	Constant		82.121	47.139	1.742	0.115
			AR	Lag 1	−0.343	0.567	−0.605	0.560
	Phase	No transformation	Numerator	Lag 0	−367.929	164.198	−2.241	0.052
	54 months preintervention	No transformation	Numerator	Lag 0	34.983	14.450	2.421	0.039
	54 months postintervention	No transformation	Numerator	Lag 0	−14.707	6.040	−2.435	0.038

Regarding new TMZ prescriptions in the PD population, the average absolute number of new prescriptions among PD patients decreased by 119 in each six-month interval (95% CI = −190.79 to −47.19, *p* = 0.005) before baseline (the first half-year of 2012) which means an average decrease of 22.2% in each preintervention half-year period. There was a temporary increase of 47.7% in TMZ initiation immediately after the EMA intervention (the second half-year of 2012) which was followed by a slight long-term decrease. Globally, the decrease in TMZ initiation slowed down and the average decrease in new prescriptions was only 6.54% (seven patients) per half-year after the EMA recommendations. Compared with baseline, no significant change in the absolute number of patients newly initiated on the drug was found on long-term after the EMA restrictions. The ARIMA model showed a negative change in the preintervention trend (105.42, 95% CI = 31.07–138.29, *p* = 0.011), combined with negative level effects at 12 months postintervention (341.11, 95% CI = 9.48–672.74, *p* = 0.045) and at all following time points. The relative 54-month effect was −119.62% (Fig. 1*B*; Table 3).

**Table 3:** ARIMA model parameters for TMZ initiation in Figure 1*B*

					Estimate	Standard error	*t* value	*p* value
ARIMA model parameters 1	Outcomes	No transformation	Constant		776.317	103.231	7.520	<0.001
			AR	Lag 1	−0.297	0.326	−0.910	0.386
	Time period	No transformation	Numerator	Lag 0	−118.993	31.738	−3.749	0.005
	Phase	No transformation	Numerator	Lag 0	−396.802	155.769	−2.547	0.031
	Interact	No transformation	Numerator	Lag 0	105.419	32.868	3.207	0.011
ARIMA model parameters 2				
	Outcomes	No transformation	Constant		776.291	103.227	7.520	<0.001
			AR	Lag 1	−0.297	0.326	−0.911	0.386
	Phase	No transformation	Numerator	Lag 0	235.690	124.116	1.899	0.090
	6 months preintervention	No transformation	Numerator	Lag 0	−118.985	31.736	−3.749	0.005
	6 months postintervention	No transformation	Numerator	Lag 0	−13.573	12.070	−1.125	0.290
ARIMA model parameters 3	
	Outcomes	No transformation	Constant		776.294	103.228	70.520	<0.001
			AR	Lag 1	−0.297	0.326	−0.911	0.386
	Phase	No transformation	Numerator	Lag 0	341.105	146.596	2.327	0.045
	12 months preintervention	No transformation	Numerator	Lag 0	−118.986	31.736	−3.749	0.005
	12 months postintervention	No transformation	Numerator	Lag 0	−13.574	12.070	−1.125	0.290
ARIMA model parameters 4				
	Outcomes	No transformation	Constant		776.297	103.228	7.520	<0.001
			AR	Lag 1	−0.297	0.326	−0.911	0.386
	Phase	No transformation	Numerator	Lag 0	446.522	172.443	2.589	0.029
	18 months preintervention	No transformation	Numerator	Lag 0	−118.987	31.736	−3.749	0.005
	18 months postintervention	No transformation	Numerator	Lag 0	−13.574	12.070	−1.125	0.290
ARIMA model parameters 5				
	Outcomes	No transformation	Constant		776.301	103.229	7.520	<0.001
			AR	Lag 1	−0.297	0.326	−0.911	0.386
	Phase	No transformation	Numerator	Lag 0	551.942	200.359	2.755	0.022
	24 months preintervention	No transformation	Numerator	Lag 0	−118.988	31.737	−3.749	0.005
	24 months postintervention	No transformation	Numerator	Lag 0	−13.574	12.070	−1.125	0.290
ARIMA model parameters 6				
	Outcomes	No transformation	Constant		776.305	103.229	7.520	<0.001
			AR	Lag 1	−0.297	0.326	−0.911	0.386
	Phase	No transformation	Numerator	Lag 0	657.365	229.590	2.863	0.019
	30 months preintervention	No transformation	Numerator	Lag 0	−118.989	31.737	−3.749	0.005
	30 months postintervention	No transformation	Numerator	Lag 0	−13.574	12.070	−1.125	0.290
ARIMA model parameters 7				
	Outcomes	No transformation	Constant		776.310	103.230	7.520	<0.001
			AR	Lag 1	−0.297	0.326	−0.910	0.386
	Phase	No transformation	Numerator	Lag 0	762.790	259.693	2.937	0.017
	36 months preintervention	No transformation	Numerator	Lag 0	−118.991	31.737	−3.749	0.005
	36 months postintervention	No transformation	Numerator	Lag 0	−13.574	12.071	−1.125	0.290
ARIMA model parameters 8				
	Outcomes	No transformation	Constant		776.315	103.231	7.520	<0.001
			AR	Lag 1	−0.297	0.326	−0.910	0.386
	Phase	No transformation	Numerator	Lag 0	868.219	290.398	2.990	0.015
	42 months preintervention	No transformation	Numerator	Lag 0	−118.992	31.737	−3.749	0.005
	42 months postintervention	No transformation	Numerator	Lag 0	−13.574	12.071	−1.125	0.290
ARIMA model parameters 9				
	Outcomes	No transformation	Constant		776.320	103.232	7.520	<0.001
			AR	Lag 1	−0.297	0.326	−0.910	0.386
	Phase	No transformation	Numerator	Lag 0	973.651	321.531	3.028	0.014
	48 months preintervention	No transformation	Numerator	Lag 0	−118.994	31.738	−3.749	0.005
	48 months postintervention	No transformation	Numerator	Lag 0	−13.574	12.071	−1.125	0.290
ARIMA model parameters 10				
	Outcomes	No transformation	Constant		776.325	103.233	7.520	<0.001
			AR	Lag 1	−0.297	0.326	−0.910	0.386
	Phase	No transformation	Numerator	Lag 0	1079.087	352.980	3.057	0.014
	54 months preintervention	No transformation	Numerator	Lag 0	−118.995	31.738	−3.749	0.005
	54 months postintervention	No transformation	Numerator	Lag 0	−13.574	12.071	−1.125	0.290

**Figure 2.**
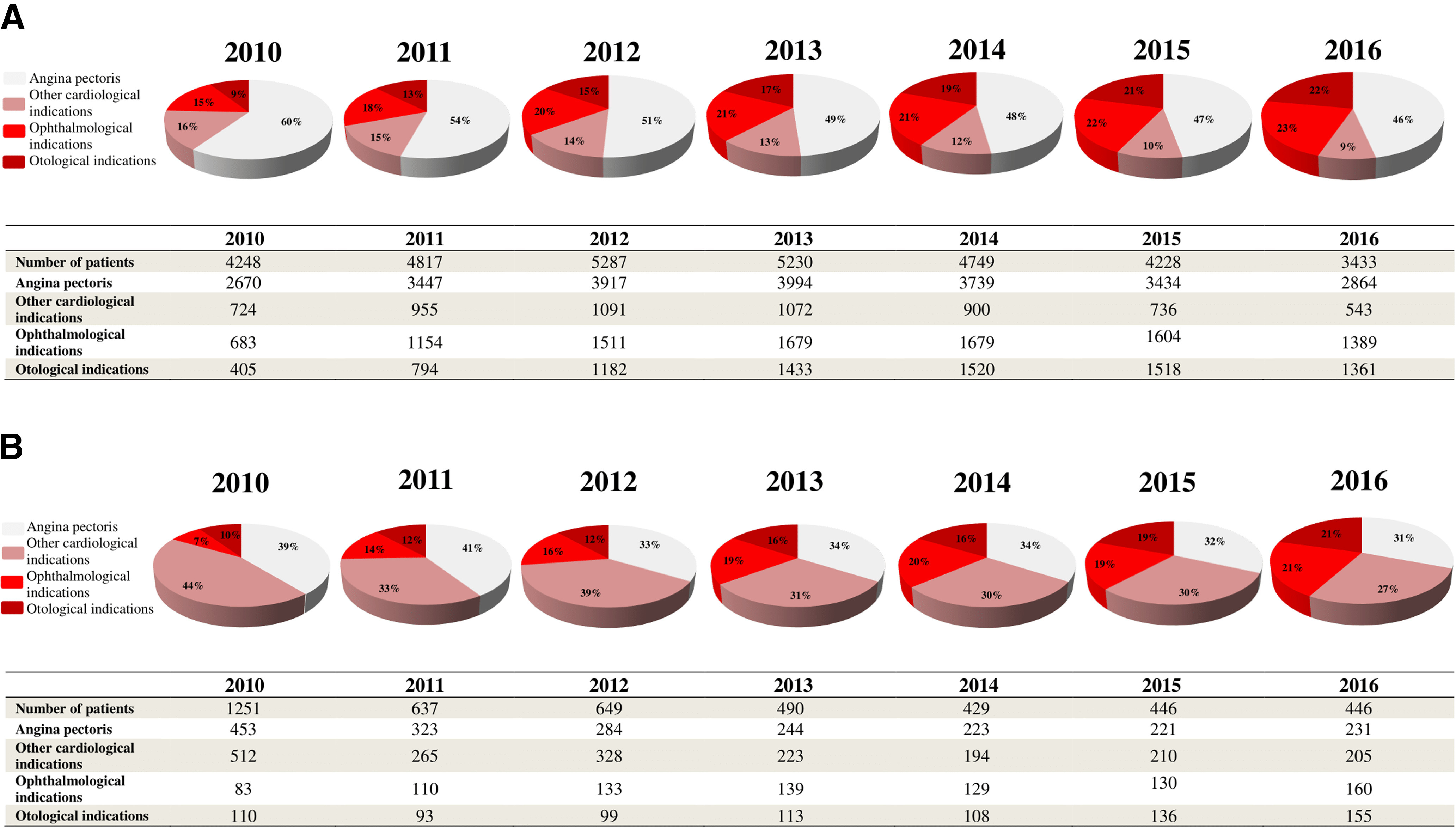
Possible indications for ongoing TMZ treatment (***A***) and new initiations on the drug (***B***) in PD from 2010 to 2016. The following categorizations were used: (1) antianginal indication (ICD-10-CM I20, on-label prescriptions); (2) other cardiological indications (ICD-10-CM I00-I99 with the exemption of ICD-10-CM I20, possibly off-label prescriptions after the EMA warning); (3) ophthalmological indications (ICD-10-CM H30-H36, definitely off-label prescriptions after the EMA warning); and (4) otological indications (ICD-10-CM H80-H83, definitely off-label indications after the EMA warning). Other non-investigated disorders might have also served as the basis of TMZ use or initiation. One patient might have had more than one diagnosis. ICD-10-CM, International Classification of WHO Diseases, 10th Revision, Clinical Modification.

Potential indications for TMZ utilization in PD are separately shown in regard to ongoing treatments and new prescriptions for every investigated year in Figure 2. The main underlying causes for ongoing TMZ use and initiation of the drug were circulatory system disorders, especially angina pectoris. However, of all detected diagnoses, the proportion of the only one on-label indication (angina pectoris) and other cardiological indications showed a slight continuous decrease over the years after the EMA recommendations for both ongoing treatment and drug initiation. In parall.el, there was a modest shift toward definitely off-label TMZ prescription. In the last investigated year, definitely off-label indications might have still been responsible for 45% and 42% of all PD cases with ongoing TMZ treatment and TMZ initiation, respectively.

## Discussion

Although the EMA recommendations on TMZ use were introduced more than seven years ago, only a single study has attempted to investigate their impact on TMZ utilization thus far (von Bredow et al., 2018). Of note, this study provides no data on possible changes in the trend of TMZ use in PD. Therefore, we aimed to explore the effectiveness of the EMA restrictions specifically focusing on the management of patients suffering from PD.

The analysis of data obtained from the National Health Insurance Fund of Hungary using an interrupted time series model revealed that the EMA procedure seemed to lead to only moderate beneficial changes in TMZ utilization among patients with PD. The main result of introducing restrictions on TMZ use is the prevention of a further increase in the use of the drug in PD. However, only a slight difference was found between the absolute numbers of PD patients treated with medications containing TMZ at the beginning and the end of the period investigated in this study. In the first half-year of 2010, there had been a total of 3950 patients with PD and concomitant TMZ use which number was reached again in the first half-year of 2015 and subsequently decreased to 3090 by the second half-year of 2016. This means a total of 21.8% decrease in overall TMZ use in PD over seven years. In the years analyzed by this study, there were ∼20,000–40,000 patients having PD in Hungary (Gustavsson et al., 2011; Szatmari et al., 2019). Consequently, 7.7–15.5% of all PD patients in our country were TMZ users after the EMA restrictions. This data sheds light on that the number of PD patients on TMZ might be still large despite the recommendation against the prescription of this drug in the PD population.

Based on our findings, the effects of the EMA procedure on TMZ use in PD mainly resulted from the increased rate of withdrawal of the drug and not the reduction in the number of new TMZ prescriptions among PD subjects. Eventually, no significant reduction appeared in the frequency of new TMZ initiations in the PD population. In the study by von Bredow et al. (2018), investigating 12 European countries, including Hungary, between 2014 and 2015, less than half (46.5%) of the asked physicians mentioned PD as a contraindication for TMZ treatment. This gap in knowledge of a great part of physicians prescribing TMZ may explain our findings in regard to new PD patient initiations on the drug.

Although the majority of patients with PD received TMZ for angina pectoris, a relatively large portion of PD patients were treated with TMZ for possibly and definitely off-label indications. These included not just ophthalmologic and otologic but also some non-anginal cardiovascular disorders. These findings are in line with the results of the study by von Bredow and colleagues that also detected frequent off-label prescription of TMZ (von Bredow et al., 2018) after the EMA procedure. A possible explanation for the frequent off-label prescription of TMZ may be that the knowledge and awareness of physicians regarding the safety communications on TMZ and the updated indications of this drug are poor which may result from the ineffective risk minimization measures and the oversight, previous experience or old habits of physicians (von Bredow et al., 2018). Another reason of this finding may be that while recent guidelines on the treatment of angina pectoris provide many alternatives for TMZ (e.g., certain β-blockers and calcium channel blockers, long-acting nitrates, and ranolazine; Knuuti et al., 2020), pharmacotherapies for tinnitus, vertigo and visual disturbances of vascular origin are very limited (Brand, 2012; von Bredow et al., 2018; Cima et al., 2019). However, the use of TMZ in these disorders is also not favored by clinical data (European Medicines Agency, 2012b). Nonpharmacological treatments (e.g., neurostimulation, tinnitus retraining therapy, sound therapy, laser photocoagulation, photodynamic therapy; Brand, 2012; Cima et al., 2019) can be good alternatives if pharmacotherapy is ineffective or not recommended. Although our results show that TMZ use in PD regarding all indications seems to be still not negligible, the EMA intervention might have affected TMZ use among PD patients in every group of prescribers, first among cardiologists found to have the most up-to-date knowledge on recent regulations on TMZ utilization (von Bredow et al., 2018), and with some delay among ophthalmologists and otolaryngologists. However, it should be also noted that TMZ could have been withdrawn also by general practitioners or neurologists.

There is increasing research into potential new indications of TMZ (Tarkin and Kaski, 2018). Potential future approval of the drug as a treatment option for further disorders might detrimentally affect achieved improvements in TMZ utilization among patients with PD if prescribers are not aware of the harmful effects of TMZ on movement disorders. Considering the minimal clinically relevant difference thresholds for the Movement Disorders Society-sponsored Unified Parkinson’s Disease Rating Scale (Horváth et al., 2015, 2017; Makkos et al., 2019), medicines containing TMZ can worsen the symptoms of PD in a clinically relevant manner (worsening of 4.0, 3.5, 10.4, and 1.2 points in the Parts I, II, III, and IV of the Movement Disorders Society-sponsored Unified Parkinson’s Disease Rating Scale), which can have a serious impact on the health-related quality of life (Pintér et al., 2020). These findings highlight the importance of compliance with the EMA recommendations in the management of PD patients having any comorbidities approved to be treated with TMZ at present and in the future.

The strength of the present study mainly lies in the used method that enabled the evaluation of changes in TMZ utilization trends among patients with PD with respect to the EMA intervention at a population level. However, for correct interpretation of the results, some potential limitations also need to be considered. First, we were able to obtain data on TMZ use only between 2010 and 2016 because of technical reasons, however, investigation of a wider period could provide a deeper knowledge of how trends in TMZ use have changed and possible underlying causes for these changes. As it can be seen in Figure 1*B*, TMZ initiation among PD patients started to decrease before the release of the EMA recommendations similarly to the increase in the withdrawal of the drug in PD. A possible explanation for this might be that literature data (Martí Massó, 2004; Martí Massó et al., 2005; Masmoudi et al., 2005; Sommet et al., 2005; Sivet et al., 2008; Commission nationale de pharmacovigilance, 2009; Réunion de la Commission d’AMM du 7 avril 2011, 2011) prompting the EMA to reevaluate the role of TMZ might have already widely disseminated among physicians before the EMA recommendations. This might also be an alternative explanation for why the present study found the EMA restrictions to be only moderately effective. However, future trials that analyze data on TMZ prescription also from the years of the release of publications (Martí Massó, 2004; Martí Massó et al., 2005; Masmoudi et al., 2005; Sommet et al., 2005; Sivet et al., 2008) and events (Commission nationale de pharmacovigilance, 2009; Réunion de la Commission d’AMM du 7 avril 2011, 2011) leading to the EMA procedure should evaluate this hypothesis. In addition, further studies providing data on the previous three years would also be helpful in obtaining a more reliable picture of the current practice with TMZ. Another issue may be that indication-linked reimbursement has not been available in regard to off-label prescription of TMZ in Hungary since the introduction of the EMA recommendations. This regulation might have had an impact on the practice of drug prescription; however, it should have not meaningfully affected the compliance with the EMA recommendations. Furthermore, the present paper did not analyze the correlation between TMZ use in PD and hospitalization or death. However, future investigations could provide additional useful data on the clinical relevance of the EMA recommendations by exploring whether TMZ treatment may lead to increased hospitalization rates and risk of death in PD. Finally, it should be also mentioned that only data representing TMZ use in Hungary was analyzed. Therefore, to judge the generalizability of our findings, further studies should be conducted in other countries where TMZ has been available. Our paper could be a good basis for planning and performing such investigations.

To conclude, the present study suggests that the EMA restrictions on TMZ use in PD are only moderately effective. The number of patients with PD on TMZ seems to remain relatively stable. Furthermore, off-label TMZ use in PD is still an unsolved problem. Possibly, another safety communication should be performed, perhaps via channels which have not previously been used (e.g., brochures, posters, advertisements on websites frequently visited by the prescribers of TMZ, mobile applications, seminars, and conferences) and with new strategies, for further education of physicians and gaining more compliance which might lead to an additional improvement in the management of PD patients.

## Synthesis

Reviewing Editor: Mikko Airavaara, University of Helsinki Decisions are customarily a result of the Reviewing Editor and the peer reviewers coming together and discussing their recommendations until a consensus is reached. When revisions are invited, a fact-based synthesis statement explaining their decision and outlining what is needed to prepare a revision will be listed below. The following reviewer(s) agreed to reveal their identity: Antti Huotarinen, Debra Ehrlich.

The authors present the effect of EMA warning on the reduction of the use of trimetazidine in patients with Parkinson's disease. Overall, this is a nice evaluation of the impact that specific EMA restrictions had on the prescribing practices of TMZ in patients with Parkinson's Disease. This highlights a potential gap in guidelines effectively translating to actual clinical practice. The article is easy to read and the message of the analysis is easy to understand. Although the presented data and analysis are relatively simple, they are important as the given warnings and recommendations are valuable only if they have an effect. Countries with single funding sources for health care combined with national registries are an important source for system level research which strengthens the manuscript. However, manuscripts simplicity underlines the need for scrutiny in presentation of the results and discussion. Below you can find detailed critic to the study that needs to be addressed.

1. One of the major conclusions made by the authors is that EMA restrictions on TMZ use in PD are only moderately effective. Since this paper only includes data from the Hungarian population, the results are not necessarily generalizable. This is an important point that should be discussed among the study limitations. If similar data from other countries is available, that would greatly strengthen the impact of this paper. If not, it is important to stress that these results may only apply to the Hungarian population.

2. It seems that the reduction of TMZ initiations started before EMA recommendations as shown by Fig 1B. As the authors state it is a limitation that data from the previous years is not available, but it seems plausible that physicians already might have become aware of TMZ related problems before EMAs recommendation. This should be discussed.

3. Based on the data provided in Fig 1B, it appears that there was already considerable reduction in initiation and increases in withdrawal of TMZ starting in 2010, years before the EMA restrictions. It would be useful to discuss possible explanations for this. Was the information regarding risks of TMZ in PD widely disseminated prior to the initiation of the EMA restrictions? If this was the case, this could suggest an alternate explanation regarding why the EMA restrictions appear to only have a modest effect on prescribing practices.

4. Figure 1B: one trendline for initiations prior to EMA recommendation is sufficient, having two seems redundant if not well argued for and justified. Only the corresponding to reported ARIMA analysis should be shown. Or at least point out which of the trendlines correspond to the analysis.

5. Looking at Figure 1B it seems that after EMA intervention the initiations and withdrawals are in balance, yet, Figure 1A shows that TMZ user is clearly declining. Why so? It should be discussed, is this due to deaths? The paper could be strengthened by showing evidence of morbidity related to trimetazidine use in PD. If available, would consider exploring whether there were increased hospitalization rates in PD patients prescribed trimetazidine.

6. Starting on row 213 strength is attributed to method but it should be attributed to availability of nationwide data from the National Health Insurance Fund of Hungary. Since the authors state that dosing would be possible to achieve as well it would be interesting to see if doses were also changed after EMA warning.

7. Discussion should consider the suitability of alternatives of trimetazidine for angina and off-label use in PD patients. Especially, since Figure 2A and B show that off-label use was proportionally higher after EMA warning. Why the authors think it is so?

8. Statistics presentation needs to be shown according to eNeuro guidelines.
